# High prevalence of severe pain is associated with low opioid availability in patients with advanced cancer: Combined database study and nationwide questionnaire survey in Japan

**DOI:** 10.1002/npr2.12448

**Published:** 2024-05-12

**Authors:** Maiko Hasegawa‐Moriyama, Yasuhide Morioka, Shinzo Hiroi, Noriyuki Naya, Yura Suzuki, Yuichi Koretaka, Erina Hara, Hiroaki Abe, Kanji Uchida, Masahiko Sumitani

**Affiliations:** ^1^ Department of Pain and Palliative Medical Sciences, Faculty of Medicine The University of Tokyo Tokyo Japan; ^2^ Medical Affairs Department Shionogi & Co., Ltd. Osaka Japan; ^3^ Data Science Department Shionogi & Co., Ltd. Osaka Japan; ^4^ Department of Anesthesiology and Pain Relief The University of Tokyo Hospital Tokyo Japan; ^5^ Department of Pain and Palliative Medicines The University of Tokyo Hospital Tokyo Japan

**Keywords:** cancer‐related pain, end‐stage cancer patients, opioid prescription, palliative care

## Abstract

**Objectives:**

Opioid availability for the palliative care of patients with advanced cancer is increasing globally. However, opioid availability remains extremely low in Japan. We investigated whether pain is appropriately controlled by low‐dose opioid prescriptions in patients with advanced cancer in Japan.

**Methods:**

A web‐based nationwide survey for caregivers from 2000 community comprehensive support care centers was performed in Japan to assess details about pain in the 30 days before patients died of end‐stage cancer. Separately, the data for opioid prescription doses and medical services in the 90 days before the death of patients with cancer were extracted from a health insurance claim database.

**Results:**

Responses from 1034 responders were retrieved and 665 patients were included. In total, 254 patients (38.2%) complained of severe‐to‐intolerable cancer‐related pain. The median cumulative prescription dose of opioids in the 90 days before patient death was 311.0 mg by oral morphine equivalent doses. Multiple regression analyses across prefectures revealed that the proportion of patients with severe‐to‐intolerable cancer‐related pain was negatively associated with the cumulative opioid consumption expressed as morphine‐equivalent doses within 90 days before death.

**Conclusions:**

The very low availability of opioids for patients with end‐stage cancer could result in high rate of severe‐to‐intolerable cancer‐related pain patients. There were several limitations in this study, and the interpretations of the findings should be carefully. However, the increase in the absolute dose of opioids could improve the palliative care framework to the pain control levels of the global standard.

## Abstract 2

We revealed very limited doses of opioids were prescribed for end‐stage cancer patients in Japan. This was the one seventeenth of the WHO standard. The limited doses were associated with high proportion of severe‐to‐intolerable end‐stage cancer pain.

## Graphical abstract

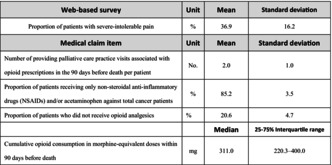

## 1 INTRODUCTION

Pain is one of the most common symptoms among patients with cancer. It decreases quality of life (QOL), and it can persist as an independent prognostic factor of survival.^1^ Pain was reported by 52.2% of the patients after curative treatment or on anticancer treatment and by 74.8% of the patients on anticancer treatment or with advanced, metastatic cancer treatment^2^

In the latest International Classification of Diseases defined by the World Health Organization (WHO), “cancer‐related pain” was appended to be defined as chronic pain attributable to either the cancer itself or its treatments. As one of the most major cancer treatment‐related pain, chemotherapy‐induced peripheral neuropathy (CIPN) sometimes sustains in years.^3^ Patients with advanced and end‐stage cancer suffer chronic pain which consists of pain by cancer itself and cancer treatment‐related pain. Opioid analgesics are the main analgesic therapies for the effective relief of cancer‐related pain, especially in patients with end‐stage cancer.^4^ Furthermore, WHO extended the indications for strong opioids to include not only severe cancer‐related pain but also mild‐to‐moderate cancer‐related pain in 2018.^5^

Nonetheless, in many nations, there are barriers to opioid availability because of public health policies, laws, or the education of healthcare professionals.^6^ To expand universal access to effective palliative care and cancer‐related pain relief, the problem of supply has been identified as one of the barriers for adequate opioid availability by the International Narcotics Control Board.^4^

Wu and colleagues reported important findings from a retrospective, nationwide database study investigating annual opioid prescription patterns from 2012 to 2017 in Taiwan. In the study, the annual cumulative opioid dosage per patient decreased over time, and the rate of opioid use among patients with cancer appeared to exhibit an extremely gradual decline.^7^ Their previous study of a nationwide survey of patients with cancer conducted in 2014 clearly revealed that one‐third of patients with cancer‐related pain conveyed dissatisfaction with pain control and prescribed analgesics, leading to significantly poor QOL‐related outcomes.^8^ It should be considered that opioid underuse and stigmas regarding opioids in Taiwan and other East Asian countries still present major challenges for cancer‐related pain management.

In Japan, the estimated total consumption of opioid analgesics did not reach the WHO's Adequacy of Consumption Measure (ACM), which is a globally accepted index used to assess opioid availability for cancer‐related pain by the WHO, in early 2010s.^9^ The significant prefectural differences in the estimated total consumption of opioids were observed.^9^ Similar to Taiwan, the estimated adequacy of opioid consumption in Japan slightly decreased from 2013 to 2015. This trend would follow that of US, where the 2016 Center for Disease Control and Prevention (CDC) guidelines recommend restricted use of opioid analgesics for chronic pain, including cancer‐related pain, to control the opioid epidemic and crisis.^10^ This US trend clearly resulted in poor outcomes for cancer‐related pain management among terminally ill patients with cancer.^11^ Hence, conflicting with the CDC guidelines, the latest US government pain management guidelines (Pain Management Best Practices Inter‐Agency Task Force, US Department of Health and Human Services, 2020) emphasize the necessity of opioid analgesics for patients with cancer, as well as the risks associated with opioid analgesics.^12^ The WHO ACM for opioids has been designed for terminally ill cancer patients who have a poor prognosis. Healthcare professionals and governments in Japan and other East Asian countries, where the global standard of adequacy of opioid availability for cancer death has not been achieved, should recognize that they have not been burdened by an opioid epidemic and crisis in the nations and region.^13^

In this study, we first aimed to investigate the current situation regarding the pain intensity of patients with end‐stage cancer receiving care from healthcare professionals in each prefecture of Japan. Next, we investigated a health insurance claim database to reveal opioid prescription patterns during the 90 days before patient death and the number or proportion of other medical services related to palliative care. Finally, we combined both results and explored factors correlated with prefectural differences in cancer‐related pain intensity and the opioid availability.

## 2 METHODS

### 2.1 Ethics

This study was performed after approval by the ethical committee of The University of Tokyo Hospital (reference 202176NI).

### 2.2 Study design and participant population

#### 2.2.1 Analysis 1

A nationwide questionnaire‐based cross‐sectional study in Japan was conducted between September 1, 2021, and November 15, 2021. Japan has more than 5000 comprehensive support care centers that formally support the elder population, disabled people, and terminally ill patients in view of health and welfare. Balancing the numbers of centers against respective prefecture populations, we selected 2000 centers nationwide in proportion to the population and sent a study invitation letter to care staffs belonging to the centers for participation in our web‐based questionnaire survey (Table 1). In the centers, qualified caregivers, nurses and medical social workers work as licensed care staffs and also unqualified office clerks work. We asked care staffs who practically take care of terminally ill cancer patients to answer the survey. We assumed the qualified caregivers as the most major answerers to the survey, but we did not specify any kinds of professions and did not gather the information on professions of answerers in the survey. We commissioned the Nippon Research Center (Tokyo, Japan) to conduct the survey. The participants were queried regarding details about their assigned patients of recent date, who died of end‐stage cancer. Immediately before answering the survey, the participants gave their consent through the Internet‐system.

**TABLE 1:** The proportion of valid to total responses for the web‐based questionnaire by prefecture.

Prefecture	No·of posted community comprehensive support care centers	No·of valid responses	No·of total responses	Proportion of valid responses (%)
Hokkaido	64	27	48	56.3
Aomori	31	19	22	86.4
Iwate	30	11	18	61.1
Miyagi	39	14	14	100.0
Akita	28	8	11	72.7
Yamagata	29	6	11	54.5
Fukushima	36	9	12	75.0
Ibaragi	44	11	26	42.3
Tochigi	36	9	13	69.2
Gunma	37	16	24	66.7
Saitama	82	33	49	67.3
Chiba	73	16	23	69.6
Tokyo	136	44	60	73.3
Kanagawa	97	45	69	65.2
Niigata	39	15	19	78.9
Toyama	29	17	23	73.9
Ishikawa	30	7	10	70.0
Fukui	27	4	9	44.4
Yamanashi	26	7	12	58.3
Nagano	37	11	19	57.9
Gifu	37	22	31	71.0
Shizuoka	51	19	29	65.5
Aichi	83	20	29	69.0
Mie	35	10	21	47.6
Shiga	32	10	15	66.7
Kyoto	41	11	18	61.1
Osaka	94	23	35	65.7
Hyogo	66	24	26	92.3
Nara	31	7	12	58.3
Wakayama	28	7	9	77.8
Tottori	25	5	8	62.5
Shimane	26	7	13	53.8
Okayama	36	18	28	64.3
Hiroshima	43	21	38	55.3
Yamaguchi	32	6	10	60.0
Tokushima	26	8	12	66.7
Kagawa	24	11	19	57.9
Ehime	31	10	15	66.7
Kochi	26	6	9	66.7
Fukuoka	63	13	16	81.3
Saga	27	14	19	73.7
Nagasaki	31	3	4	75.0
Kumamoto	35	20	31	64.5
Oita	30	11	18	61.1
Miyazaki	29	8	14	57.1
Kagoshima	34	10	14	71.4
Okinawa	32	12	15	80.0
Total/average	2000	665	1000	66.5

The survey included 45 questionnaire items about palliative medicine topics including physical symptoms (e.g., pain, fatigue, nausea, and so on), psychological distress, disclosure of cancer diagnosis, place of end‐of‐life care and dying‐in‐place, and utilization of social welfare services. We focused on cancer‐related pain, which is evaluated by the item “Please answer your assigned cancer patient's physical conditions during the end‐of‐life care. How do you assess your assigned patient's pain one week before cancer death.” Pain intensity was assessed by the five‐point Likert scale (i.e., none, mild, moderate, severe, and intolerable). We did not ask our participants to answer their assigned patient's pain in distinguishing pain by cancer itself from cancer treatment‐related pain, and then these results were aggregated by prefecture.

#### 2.2.2 Analysis 2

To analyze data related to palliative care practice and opioid prescription doses in the 90 days before cancer death, we used the Japanese health insurance claim database, which has accumulated receipts in various settings (inpatient, outpatient, dispensing). As the largest health insurance society in Japan, the database covers 25% of the Japanese population provided by the Japan Medical Data Center (JMDC).

Because this database was commercially provided with data anonymization, the need for informed consent from each participant was therefore waived. The data of patients aged >18 years with a diagnosis of cancer (ICD‐10: C00‐D09) within 6 months before death from September 2011 to August 2021 who had records for 90 days before death were included.

Deidentified datasets including diagnosis, demographic characteristics, medication, and claims were retrieved. Patient demographics are described in Table S1. We focused on 25 claim variables associated with palliative medical services within 90 days before death. The mean prescription prevalence (%) of opioids for patients over 90 days before death was shown with respect to each prefecture. The median cumulative prescribed doses of opioids per patient over 90 days before death was retrieved and then converted to morphine equivalents. In Japan, several opioid medicines are available for managing cancer‐related pain. The lates WHO guidelines^5^ recommend any opioid medicines, but especially remark the doses of opioids for relieving the patients' pain to an acceptable level. We therefore did not gather information about opioid medicines from the database, but only gathered the doses of opioids which are converted and determined to oral morphine (namely, morphine‐equivalent doses) based on the Japanese calculations,^14^ which is approximately same as the WHO guidelines.^5^

### 2.3 Statistical analysis

As reflecting symptoms as a quality indicator for palliative care, the results of the web‐based questionnaire and those of epidemiological receipt database were analyzed together, to explore the cause of severe‐to‐intolerable pain and opioid prescription doses in the following manner. Two multiple regression analyses across 47 prefectures were performed. In the first analysis, multiple regression analysis was performed targeting the proportion of patients with severe‐to‐intolerable pain regarded as an outcome. In the second analysis, multiple regression analysis was performed targeting opioid prescription as an outcome. Any variables related to palliative care services, among 25 claim variables, were arbitrarily selected according to clinical experiences and regarded as independent variables in the construction of multiple regression models. We explored and constructed multiple regression models over and over again, which plausibly reflect the current situations of terminally ill cancer patients and opioid availability in Japan and can inspire improving the end‐of‐life care before cancer death. Then, elimination was performed to delete variables with the lowest contribution and those with multicollinearity from 25 claim variables associated with palliative medical services within 90 days before death until the final model was obtained. In the final models, backward elimination strategies were applied for 11 selected services to predict severe‐to‐intolerable cancer‐related pain intensity, and 10 variables for opioid dosages were regarded as independent variables among 25 imputed datasets to avoid over‐selection of the variables (variables significant at *p* < 0.05 were retained). The two multiple regression analyses adopted the 10 common variables, and the cumulative opioid dosages were further added the 11th independent variable in the analysis for predicting severe‐to‐intolerable pain. Analyses were performed using SPSS software version 22 (IBM Corp, Armonk, NY, USA). *p* < 0.05 was considered significant.

## 3 RESULTS

### 3.1 Analysis 1

Data from 1034 responders from 2000 comprehensive support centers nationwide were retrieved, and 1000 completed questionnaires for patients who died of end‐stage cancer were considered valid (Table 1). Of these, 665 answers excluding the answers “I don't want to answer,” “unknown,” and “not applicable” were used for analysis. The demographics of their assigned patients such as sex, age, and cancer type are described in Table S2. The ratios of valid to total responses for the web‐based questionnaire by 47 prefectures are described in Table 1. Questionnaire analysis by prefecture revealed that 486 of 665 patients (73·1%) with end‐stage cancer complained of moderate‐to‐intolerable cancer‐related pain within 30 days before death. In addition, 254 of 665 patients (38.2%) complained of severe‐to‐intolerable cancer‐related pain. However much varied valid response rates were observed among prefectures, we presented the results of the prefecture‐by‐prefecture analysis for reference sake (Figure S1). The average proportion of patients with severe‐to‐intolerable cancer‐related pain by prefecture was 36.9 ± 16.2% (mean ± SD) (Table 2).

**TABLE 2:** Descriptive analysis of data from the web‐based survey and medical claims for palliative care services in the 47 prefectures.

	Unit	Mean	Standard deviation
Web‐based survey			
Proportion of patients with severe‐intolerable pain	%	36.9	16.2
Medical claim item			
Number of providing palliative care practice visits associated with opioid prescriptions in the 90 days before death per patient	No.	2.0	1.0
Number of basic palliative care practice visits unrelated to opioid prescriptions in the 90 days before death per patient	No.	0.1	0.05
Proportion of patients receiving only nonsteroidal anti‐inflammatory drugs (NSAIDs) and/or acetaminophen against total cancer patients	%	85.2	3.5
Proportion of patients who received any anti‐constipation drugs	%	74.6	4.7
Proportion of patients who did not receive opioid analgesics	%	20.6	4.7
Proportion of patients with basic palliative care practice visits unrelated to opioid prescriptions in the 90 days before death per patient to those related to opioid prescription	%	5.5	2.5
Proportion of patients who received specialized palliative care including opioid prescriptions in the inpatient setting	%	14.4	7.1
Proportion of patients receiving opioid analgesics which are prescribed by specialized palliative physicians in the outpatient setting	%	1.1	1.1
Proportion of basic palliative care practice visits associated with opioid analgesics prescribed by physicians not trained in basic palliative care	%	18.6	12.1
Proportion of basic palliative care practice visits associated with opioid analgesics prescribed by physicians “not” trained in basic palliative care	%	22.2	6.3
		**Median**	**25%–75% Interquartile range**
Cumulative opioid consumption in morphine‐equivalent doses within 90 days before death	mg	311.0	220.3–400.0

### 3.2 Analysis 2

**FIGURE 1:**
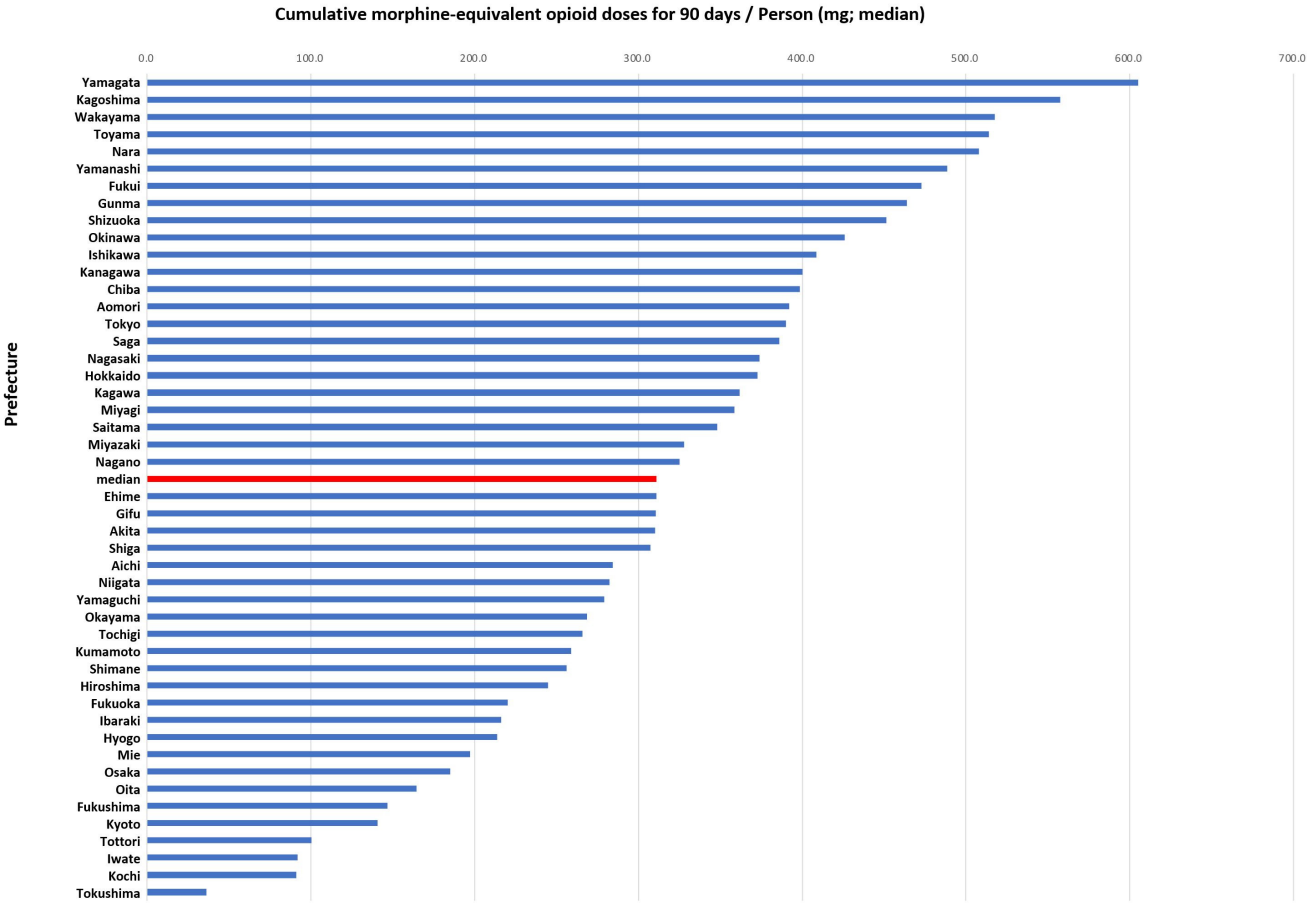
Cumulative morphine‐equivalent opioid prescription doses in the 90 days before death among patients with end‐stage cancer in each prefecture (mg, median).

Patient demographics are described in Table S1. Representative claims variables from 25 items of the Japanese health insurance claim database that were clinically associated with palliative medical services within 90 days before death are listed in Table 2. The prescription prevalence of opioids was 80.1 ± 4.7% across 47 prefectures. The range of the prevalence was not narrow (Tokushima 67.1%; Aomori prefecture, 91.2%; Table S3). The median dose of cumulative morphine‐equivalent opioid prescription over the 90‐day period per person by prefecture in Japan was 311.0 mg (interquartile range, 220.3–400.0; Table 2 and Figure 1). The difference in the minimum and maximum median cumulative morphine‐equivalent opioid prescription doses by prefecture was 16.7‐fold (Tokushima prefecture, 36.3 mg; Yamagata prefecture, 605 mg). The mean number of palliative care practice visits associated with opioid prescriptions in the 90 days before death was 2.0 ± 1.0 (mean ± SD) per patient (Table 2). The proportion of patients with at least one basic palliative care practice visit unrelated to opioid prescriptions within 90 days before death was 0.1 ± 0·05%. The proportion of patients who received opioid analgesics under the outpatient setting by specialized palliative physicians was 1.1 ± 1.1%.

### 3.3 Results of multiple regression analyses using combined data from analyses 1 and 2

The proportion of patients who received no opioid analgesics was negatively associated with the proportion of patients with severe‐to‐intolerable pain in the 30 days before death (Table 3). Similarly, the cumulative opioid consumption (morphine‐equivalent doses) within 90 days before death was negatively associated with the proportion of patients with severe‐to‐intolerable pain within 30 days before death.

**TABLE 3:** Multiple regression analysis of patients with severe‐to‐intolerable cancer‐related pain.

	Unstandardized coefficient		Standardized coefficient	t	*p*	Collinearity statistics	
	B	SE	Beta			Tolerance	VIF
Constant	164.2	55.6		3.0	0.005		
Number of providing palliative care practice visits associated with opioid prescriptions in the 90 days before death per patient	3.7	2.5	0.2	1.5	0.1	0.8	1.2
Number of providing the basic palliative care practice visits unrelated to opioid prescriptions in the 90 days before death per patient	−80.1	48.1	−0.3	−1.7	0.1	0.8	1.2
Proportion of patients who received any anti‐constipation drugs	−1.0	0.6	−0.3	−1.6	0.1	0.6	1.8
Proportion of patients who did not receive opioid analgesics	−1.7	0.7	−0.5	−2.4	0.02	0.4	2.3
Cumulative opioid consumption in morphine‐equivalent doses within 90 days before death (mg)	−0.05	0.02	−0.4	−2.2	0.03	0.6	1.8

Abbreviations: B, partial regression coefficient; beta, standardized partial regression coefficient; SE, standard error for B; VIF, variance inflation factor.

The second multiple regression analysis revealed that the total number of basic palliative care practice visits per patient unrelated to opioid prescriptions in the 90 days before death was negatively associated with the opioid prescription dose during in this period (Table 4).

**TABLE 4:** Multiple regression analysis of opioid prescription doses in the 90 days before patient death.

	Unstandardized coefficient		Standardized coefficient	t	*p*	Collinearity statistics	
	B	SE	Beta			Tolerance	VIF
Constant	794.8	97.4		8.2	<0.001		
Number of basic palliative care practice visits unrelated to opioid prescriptions in the 90 days before death per patient	−924.6	421.7	−0.4	−2.2	0.03	0.3	3.0
Proportion of patients who did not receive opioid analgesics	−20.0	2.9	−0.7	−7.0	<0.001	0.8	1.2
Proportion of patients who received opioid analgesics prescribed by specialized palliative physicians in the outpatient setting	57.8	12.4	0.5	4.7	<0.001	0.9	1.2
Proportion of patients with basic palliative care practice visits unrelated to opioid prescriptions in the 90 days before death per patient to those related to opioid prescription	8.6	8.2	0.2	1.0	0.3	0.4	2.8
Proportion of basic palliative care practice visits associated with opioid analgesics prescribed by physicians “not” trained in basic palliative care	−2.5	2.2	−0.1	−1.1	0.3	0.8	1.3

Abbreviations: B, partial regression coefficient; beta, standardized partial regression coefficient; SE, standard error for B; VIF, variance inflation factor.

## 4 DISCUSSION

### 4.1 Main findings

This study revealed that the proportion of end‐stage patients with severe‐to‐intolerable cancer‐related pain exceeds 30% in Japan. Although about 80% of end‐stage patients received opioids, the median cumulative opioid prescription dose for terminally ill patients over the 90 days before death was only 311.0 mg oral morphine equivalents (Figure 1). Our findings suggest that in Japan cancer‐related pain is not still appropriately controlled by low‐dose opioid prescriptions for patients with advanced cancer.

The ACM indicates the oral morphine‐equivalent dosage of opioid analgesics required for pain relief in terminally ill patients with cancer in milligrams per capita based on the assumption that 80% of such patients require an oral morphine‐equivalent dose of 75 mg/day over the 90 days before death^15^ Namely, a total opioid dose of 5400 mg per person is recommended over the last 90 days before death by WHO. However, this Japanese real‐world database analysis revealed the prescription doses of opioids for patients with end‐stage cancer do not reach this at all. A previous study using another Japanese claim database reported that the daily opioid dose was less than 60 mg oral morphine equivalents in most popular (30%–40%) of patients with end‐stage cancer in the last 30 days before death although tangible data of prescribed opioid doses were not shown.^14^ Elderly patients required less opioid doses than younger patients.^16^ Our present finding that about 80% of end‐stage patients received opioids over the 90 days before death was relatively larger than both elderly and younger participants in the previous study (prescription prevalence, 60–70%),^16^ because most of our participants were less than the age of 65. Referencing to the ACM recommendation by WHO and also considering such younger population of end‐stage cancer patients in this study, it is possible that the opioid prescription dose is insufficient, resulting in severe‐to‐intolerable cancer‐related pain in Japan.

Aiming to take particular actions to decrease the incidence of severe‐to‐intolerable cancer‐related pain and improving the ACM, we investigated a health insurance claim database using multiple analyses and interpreted the present findings toward the objectives. The proportion of patients who did not receive opioid analgesics was negatively associated with proportion of patients with severe‐to‐intolerable cancer‐related pain (Table 2). It appears that the initiation of opioids in patients with cancer‐related pain is well distributed. The cumulative opioid consumption in morphine‐equivalent doses within 90 days before death was negatively associated with the incidence of patients with severe‐to‐intolerable cancer‐related pain (Table 2), indicating that a higher median cumulative dose of opioids resulted in fewer patients with severe‐to‐intolerable cancer‐related pain. Considering the high incidence of patients with severe‐to‐intolerable cancer‐related pain and the low doses of opioid prescriptions in Japan, a higher opioid doses should be appropriately prescribed to these patients in terms of appropriate pain management.

The opioid prescription dose depends on certified palliative care in the outpatient setting, but not the inpatient setting (Table 4). In Japan, the interdisciplinary palliative care support has been consolidated under the inpatient setting in Japan. Our previous preliminary study demonstrated the outpatient medical expenditure per day for hypertension and diabetes, which are representative of primary care practice, were associated with prefectural differences in the adequacy of opioid availability in Japan.^9^ Encouraging and educating physicians to play a more important role in cancer‐related pain management would contribute to overcoming the two aforementioned challenges.

Our goal is to decrease the number of patients with severe‐to‐intolerable pain in Japan. For appropriate pain assessment and opioid prescription, the lack of training and awareness of healthcare professionals has been identified as the primary barrier for adequate opioid availability.^10^ Education to balance the advantages of improving adequate opioid availability with preventing opioid addiction is essential for realizing the benefits of opioid analgesics and minimizing their drawbacks to improve QOL among terminally ill patients with cancer. Opioid availability is extremely low in East Asian countries including Japan as well as Eastern Europe compared with that in other advanced countries in Western Europe, the United States, and Canada.^13^ There might be ingrained factors affecting pain control practices in different global regions. Berterame et al.^17^ identified impediments to opioid use, including the absence of training and awareness among healthcare professionals, cultural attitudes, fear of dependence, fear of diversion, and onerous regulation. Our study suggests that trained palliative physicians, preferably palliative care team members, are required for appropriate opioid prescription to control severe‐to‐intolerable cancer‐related pain at the level of the global standard in patients with end‐stage cancer.

### 4.2 Study limitations

There are several limitations in combining the results of two analyses. First, our methodology in pain assessment should be raised as a limitation. No symptoms were obtained from the health insurance claims database. Instead, objective indicators were obtained using a questionnaire survey targeting care staffs closest to patients at the end stage of cancer. The valid response rates were very varied among prefectures, and therefore this requires consideration whether each prefectural response really represents the situation around the patients at the end stage of cancer. And, we asked care staffs with any kinds of health professions to answer the survey but did not gather the information on their professions. Most pain and other symptoms intensities of cancer patients were adequately assessed by health care providers as same as the patients, but some were sometimes underestimated and the other sometimes overestimated.^18^ Although differences in attitudes to and assessment of the physical symptoms intensities among varied health care providers are still controversial,^19^ nurses and caregivers generally perform pain assessment and documentation rightly.^20^ Second, the health insurance claims database used in this study included patients with cancer employed by companies or their relatives, who were aged 65 years or less. Since approximately 75% of cancer patients appear to be over 65 years of age, the present findings might different from the Japanese current situations. Focusing age distribution of cancer patients, the present interpretation of extremely low opioid availability in Japan is consistent because elderly patients with cancer usually require less opioid doses for their cancer‐related pain management. Third, a medical fee for basic palliative care practices prescribing opioid analgesics has been claimed by physicians regardless of their training in basic palliative care before 2016. The differences in pain intensity and opioid prescription doses between trained and “untrained” physicians could not be clarified in this study. However, a recent study using another Japanese claim database revealed that the proportion of patients with end‐stage cancer who received opioid prescriptions increased from 2016 to 2017, and this increase was especially notable among patients staying in palliative wards compared with general hospitals.^16^ This suggests that opioid prescriptions provided by trained palliative physicians are important for opioid dose adjustment. And finally, in addition to these limitations, we should consequently raise it as one of limitations that two cohorts in combining analyzed were quite different. Based on this limitation, we should very carefully interpret the present findings. Nevertheless, we could consider that the findings achieve a certain appropriateness because the combined analysis clearly demonstrated that the very low availability of opioids for patients with end‐stage cancer is associated with high rate of severe‐to‐intolerable cancer‐related pain.

## 5 CONCLUSION

Although several limitations were raised to carefully interpret our present findings, the present study demonstrated that considerable proportion of end‐stage cancer patients suffer severe‐to‐intolerable pain and using the real‐world database extremely low opioid doses were prescribed immediately before cancer death, which do not reach the WHO recommendation doses at all. Across 47 prefectures, there were significant variations in the proportion of severe‐to‐intolerable cancer patients and the opioid doses.

## AUTHOR CONTRIBUTIONS

YM, SH, NN, and MS contributed to study concept and design. YM, NN, YS, and YK contributed to data collection. EH and MS assisted MHM with data analysis in Analysis 1. YS and YK contributed to data analysis. MHM wrote the initial draft of the manuscript. YM, SH, NN, YS, YK, and MS contributed to manuscript preparation. HA reviewed the statistical analysis. KU reviewed the overall content of the manuscript. MS contributed to funding acquisition. All authors are fully responsible for the overall content for the work and the conduct of the study. All authors had access to the data and the decision to publish the study. All authors reviewed the manuscript and approved the submitted final version.

## CONFLICT OF INTEREST STATEMENT

*Declaration of funding*: Shionogi & Co., Ltd. had a role in the collection of data from the JMDC database. Nippon Zoki Pharmaceutical Inc. and Heartfelt Inc. had no role in the collection, analysis, and interpretation of data; the writing of the manuscript; and the decision to submit the manuscript for publication. All authors have full access to all study data and take responsibility for the decision to submit the study for publication. *Declaration of financial/other relationship*: The department to which MH‐M belongs is supported by Shionogi & Co., Ltd., Nippon Zoki Pharmaceutical, Heartfelt, and Aiwa Hospital. KU has a collaborative research agreement including research funding with the Nihon Kohden Corporation (Tokyo, Japan) and Nipro Corporation (Osaka, Japan) on topics unrelated to the present study. MS received funding for this study from Shionogi & Co., Ltd., Nippon Zoki Pharmaceutical, Heartfelt Inc. and Aiwa Hospital and payments for lectures from Daiichi‐Sankyo and GlaxoSmithKline. YM, SH, NN, YS, and YK are employees of Shionogi & Co., Ltd. NN and YS are stockholders of Shionogi & Co., Ltd. All other authors declare no competing interests.

## ETHICS STATEMENT

Approval of the Research Protocol by an Institutional Review Board: This study was performed after approval by the ethical committee of The University of Tokyo Hospital (reference 202176NI).

Informed Consent: The participants gave their consent through the Internet system to participate in Analysis 1. In Analysis 2, we used the claim database with data anonymization and the need for informed consent from each participant was waived.

Registry and the Registration No. of the Study/Trial: N/A.

Animal Studies: N/A.

## Supporting information


Figure S1.

Table S1.

Table S2.

Table S3.

## Data Availability

We cannot provide raw data being freely available because we did not obtain agreements to release the data from the study participants and the supplier and because our ethical approval did not include the release. Instead, the datasets used and/or analyzed during this study are completely available from the corresponding author for collaborative research purposes upon reasonable request.
