# Supplementary figures and images for: High prevalence of severe pain is associated with low opioid availability in patients with advanced cancer: Combined database study and nationwide questionnaire survey in Japan
Source: Neuropsychopharmacol Rep. 2024 May 12;44(3):502–11. doi: 10.1002/npr2.12448 (PMC11544452; doi:10.1002/npr2.12448)

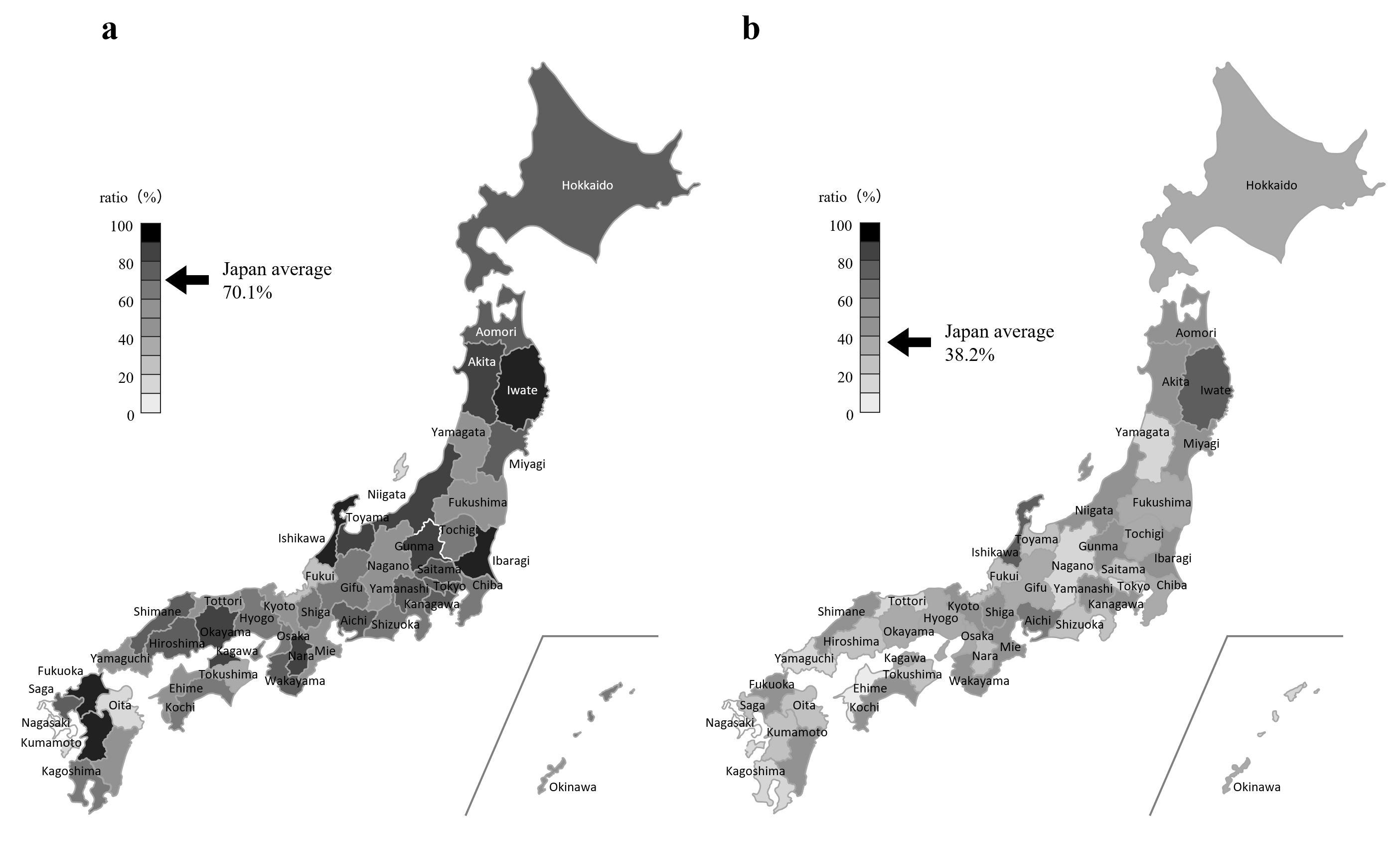

Supplement: Supplementary file 1 — Figure S1. [file NPR2-44-502-s004.tiff]
