# Supplementary material for: High prevalence of severe pain is associated with low opioid availability in patients with advanced cancer: Combined database study and nationwide questionnaire survey in Japan
Source: Neuropsychopharmacol Rep. 2024 May 12;44(3):502–11. doi: 10.1002/npr2.12448 (PMC11544452; doi:10.1002/npr2.12448)
Supplement: Supplementary file 2 — Table S1. [file NPR2-44-502-s001.docx]

**Supplemental Table 1. Patient demographics extracted from the Japanese health insurance claim database.**

| **Sex** | **Male** | 12,433 (57.3) |
| --- | --- | --- |
|  | **Female** | 9,270 (42.7) |
| **Age (years)** |  | 58.0 ± 10.0 |
| **Cancer type** | **Lung cancer** | 3,750 (17.3) |
|  | **Breast cancer** | 2,403 (11.1) |
|  | **Esophageal cancer** | 1,079 (5.0) |
|  | **Gastric cancer** | 2,562 (11.8) |
|  | **Colon cancer** | 3,130 (14.4) |
|  | **Liver cancer** | 1,378 (6.4) |
|  | **Pancreas cancer** | 2,444 (11.3) |
|  | **Kidney/urinary tract cancer** | 603 (2.8) |
|  | **Prostate cancer** | 451 (2.1) |
|  | **Uterine cancer** | 979 (4.5) |
|  | **Ovarian cancer** | 924 (4.3) |
|  | **Head and neck cancer** | 968 (4.5) |

Age is presented as the mean ± standard deviation. All other data are presented as number (%).
