# Supplementary material for: High prevalence of severe pain is associated with low opioid availability in patients with advanced cancer: Combined database study and nationwide questionnaire survey in Japan
Source: Neuropsychopharmacol Rep. 2024 May 12;44(3):502–11. doi: 10.1002/npr2.12448 (PMC11544452; doi:10.1002/npr2.12448)
Supplement: Supplementary file 3 — Table S2. [file NPR2-44-502-s002.docx]

**Supplemental Table 2. Patient demographics in the nationwide questionnaire survey**

|  |  | **Number of patients** | **Proportion (%)** |
| --- | --- | --- | --- |
| **Sex** | **Male** | 544 | 54.4 |
|  | **Female** | 429 | 42.9 |
|  | **Refused to respond** | 12 | 1.2 |
|  | **No response** | 15 | 1.5 |
| **Age (years)** | **< 20** | 1 | 0.1 |
|  | **20-29** | 1 | 0.1 |
|  | **30-39** | 4 | 0.4 |
|  | **40-49** | 33 | 3.3 |
|  | **50-59** | 57 | 5.7 |
|  | **60-69** | 134 | 13.4 |
|  | **>70** | 745 | 74.5 |
|  | **Refused to respond** | 14 | 1.4 |
|  | **No response** | 11 | 1.4 |
| **Cancer type** | **Lung cancer** | 283 | 28.3 |
|  | **Breast cancer** | 52 | 5.2 |
|  | **Esophageal cancer** | 34 | 3.4 |
|  | **Gastric cancer** | 122 | 12.2 |
|  | **Colon cancer** | 139 | 13.9 |
|  | **Liver cancer** | 115 | 11.5 |
|  | **Pancreas cancer** | 144 | 14.4 |
|  | **Bladder cancer** | 32 | 3.2 |
|  | **Prostate cancer** | 35 | 3.5 |
|  | **Cervical cancer** | 25 | 2.5 |
|  | **Hematopoietic cancer** | 30 | 3.0 |
|  | **Others** | 146 | 14.6 |
|  | **Unknown** | 16 | 1.6 |
|  | **Refused to respond** | 8 | 0.8 |
|  | **No response** | 19 | 1.9 |
