# Supplementary material for: High prevalence of severe pain is associated with low opioid availability in patients with advanced cancer: Combined database study and nationwide questionnaire survey in Japan
Source: Neuropsychopharmacol Rep. 2024 May 12;44(3):502–11. doi: 10.1002/npr2.12448 (PMC11544452; doi:10.1002/npr2.12448)
Supplement: Supplementary file 4 — Table S3. [file NPR2-44-502-s003.docx]

**Supplementary Table 3. Prescription prevalence of opioids across 47 prefectures**

| **Prefecture** | **Prevalence (%)** |
| --- | --- |
| Hokkaido | 81.2 |
| Aomori | 91.2 |
| Iwate | 67.9 |
| Miyagi | 86.4 |
| Akita | 81.4 |
| Yamagata | 86.3 |
| Fukushima | 75.0 |
| Ibaragi | 78.7 |
| Tochigi | 79.3 |
| Gunma | 81.9 |
| Saitama | 78.2 |
| Chiba | 81.4 |
| Tokyo | 82.5 |
| Kanagawa | 82.4 |
| Niigata | 81.1 |
| Toyama | 83.2 |
| Ishikawa | 84.7 |
| Fukui | 71.9 |
| Yamanashi | 86.7 |
| Nagano | 77.4 |
| Gifu | 80.7 |
| Shizuoka | 82.1 |
| Aichi | 81.0 |
| Mie | 74.7 |
| Shiga | 80.0 |
| Kyoto | 76.5 |
| Osaka | 76.4 |
| Hyogo | 75.7 |
| Nara | 78.4 |
| Wakayama | 77.1 |
| Tottori | 71.4 |
| Shimane | 82.2 |
| Okayama | 82.6 |
| Hiroshima | 79.3 |
| Yamaguchi | 80.0 |
| Tokushima | 67.1 |
| Kagawa | 80.7 |
| Ehime | 78.0 |
| Kochi | 72.2 |
| Fukuoka | 76.0 |
| Saga | 84.4 |
| Nagasaki | 79.8 |
| Kumamoto | 77.8 |
| Oita | 78.1 |
| Miyazaki | 74.4 |
| Kagoshima | 82.2 |
| Okinawa | 83.0 |
| Total  (mean ± standard deviation) | 80.1±4.7 |
